# Transcriptional Profiles of *Treponema denticola* in Response to Environmental Conditions

**DOI:** 10.1371/journal.pone.0013655

**Published:** 2010-10-27

**Authors:** Ian McHardy, Caroline Keegan, Jee-Hyun Sim, Wenyuan Shi, Renate Lux

**Affiliations:** 1 Department of Microbiology, Immunology, and Molecular Genetics, University of California Los Angeles, Los Angeles, California, United States of America; 2 School of Dentistry, University of California Los Angeles, Los Angeles, California, United States of America; Instituto Butantan, Brazil

## Abstract

The periodontal pathogen *T. denticola* resides in a stressful environment rife with challenges, the human oral cavity. Knowledge of the stress response capabilities of this invasive spirochete is currently very limited. Whole genome expression profiles in response to different suspected stresses including heat shock, osmotic downshift, oxygen and blood exposure were examined. Most of the genes predicted to encode conserved heat shock proteins (HSPs) were found to be induced under heat and oxygen stress. Several of these HSPs also seem to be important for survival in hypotonic solutions and blood. In addition to HSPs, differential regulation of many genes encoding metabolic proteins, hypothetical proteins, transcriptional regulators and transporters was observed in patterns that could betoken functional associations. In summary, stress responses in *T. denticola* exhibit many similarities to the corresponding stress responses in other organisms but also employ unique components including the induction of hypothetical proteins.

## Introduction

Invasive oral spirochetes including *Treponema denticola*, the model organism for this notoriously difficult to cultivate phylum, are believed to contribute to periodontal disease. They are frequently isolated from diseased sites and their abundance is highly correlated with periodontal pocket depth [1]. *T. denticola* is often found integrated into an anaerobic community of bacteria that includes *Tannerella forsythia* and *Porphyromonas gingivalis* [2]. In addition to surviving in gingival crevicular fluid and invading gingival tissue, spirochetes have been suggested to enter the bloodstream and contribute to atherosclerotic plaque which ultimately can lead to endocarditis or myocarditis [3], [4], [5], [6].

Stress responses are essential for adaptation, survival and propagation of all bacteria, pathogenic or otherwise. Bacteria residing in the oral cavity face particularly egregious fluctuations in nutrients, temperature, osmolarity, pH, and oxygen within their environment. Differential production of proteins associated with stress responses has been demonstrated in a number of oral species upon temperature and oxygen stress [7]; however, little is known regarding the corresponding changes in *T. denticola*. Heat shock responses of oral bacteria are necessitated by febrility and host consumption of hot substances. Transcriptional responses to heat shock in other bacteria usually involve induction of so-called heat shock proteins (HSPs), which are comprised of chaperones and ATP-dependent proteases that refold and degrade misfolded cellular proteins, respectively. GroES, GroEL, GrpE, DnaJ, and DnaK comprise the most highly conserved and easily recognizable chaperones. Commonly identified ATP-dependent proteases include Lon, FstH, DegP, and various Clp proteases. The presence of GroEL and DnaK in *T. denticola* was demonstrated via pulse chase and western blot analysis, though their induction during heat stress was not conclusive [8], [9]. The anaerobic *T. denticola* resides in gingival crevicular fluid where residual oxygen concentrations can exceed 10% [10]. This spriochete can metabolize oxygen to a certain extent [11] and genome analysis suggested the presence of an alkyl hydroperoxide reductase peroxiredoxin, a desulfoferrodoxin neelaredoxin and Nox for tolerating oxygen stress [12]. Differential production of proteins related to oxygen metabolism, however, was never confirmed for *T. denticola*. The ability to tolerate osmotic downshift, which is encountered upon transition from the isotonic gingival crevicular fluid to hypotonic saliva [13], constitutes another important, albeit neglected, stress response for periodontal bacteria. Certain non-oral microorganisms respond to hypo-osmotic stress via aquaporins and mechano-sensitive membrane channels that help regulate intracellular solute concentrations [14], [15]. In addition, a number of gram negative species produces periplasmic glucans that aid in osmotic tolerance [16].

Although *T. denticola* has been identified in atherosclerotic plaque and is hypothesized to remain metabolically active in blood, studies confirming its ability to survive in this distinct environment that would require immune evasion mechanisms are still lacking [3], [4], [5], [6]. While FhbB and Dentilisin have been implicated in immune evasion [17], [18], the genetic components of *T. denticola* stimulated in response to blood exposure are not known. Identification of such genes could be useful in identifying mechanisms of pathogenesis. Since stress response genes play critical roles in the virulence of other pathogenic bacteria, the analysis of responses to environmental stresses could add to our understanding of *T. denticola* mediated pathogenic events [7], [19].

In this study, we investigated the transcriptional profiles of *T.denticola* upon heat shock, oxygen stress as well as osmotic downshift and extrapolated genes that could comprise a core stress response. We also examined the differential gene expression in the presence of blood to highlight genes that could be relevant during infection.

## Materials and Methods

### Bacterial strain and growth conditions

*Treponema denticola* ATCC 35405 was cultivated anaerobically (5% CO_2_, 5% H_2_ and 90% N_2_) at 37°C in TYGVS medium [20]. Exponentially growing cells (OD_600_ ∼0.5) were used for all experiments unless otherwise specified. Oxygen stress was induced by shaking 10 ml cultures (∼8×10^9^ cells) in an aerobic shaker at 250 rpm at 37°C. Heat stress was applied by shifting tightly sealed 10 ml anaerobic cultures from 37°C to a 42°C water bath. Osmotic downshift was induced by diluting anaerobic 10 ml cultures into 30 ml pre-reduced, pre-warmed ddH_2_O, followed by an additional anaerobic incubation at 37°C. Blood exposure was performed with 10 ml cultures that were incubated in the presence of 20% fresh, pre-reduced, pre-warmed, defibrinated sheep blood (Colorado Serum Company) in TYGVS at 37°C. All treatments for microarray experiments were performed for 1 hour with five replicates each except for blood exposure which was performed in quadruplicate. Control samples were prepared from the same original cultures as the experimental samples and were grown under anaerobic conditions at 37°C. Cells in the control samples for osmotic downshift and blood exposure were diluted at identical ratios in fresh, reduced medium to adjust for differential gene regulation triggered by dilution effects. *Escherichia coli* strain DH5α was used for cloning experiments and propagated in LB medium.

### RNA extraction and purification

Immediately following the treatments described above, samples (∼8×10^9^ cells) were pelleted at 4,600×g for 10 minutes at room temperature. RNA was harvested using Trizol® Plus Reagent (Invitrogen Corp., CA, USA) according to the manufacturer's provided protocol. To account for potential RNA contamination, sheep blood was subjected to the same RNA extraction portocol. Only a small amount of RNA was extracted from blood and added to the appropriate control samples using extraction volume as the normalizing factor. No cross-hybridization of this RNA was observed. Extracted RNA was treated with DNAse I (Ambion) to remove traces of genomic DNA. A lack of contaminating DNA was confirmed by performing PCR on RNA samples using TDE0937 and TDE0670 primer pairs (Table 1). RNA samples were then further purified using the RNeasy Mini kit (Qiagen) according to the manufacturer's protocol.

**Table 1:** Primers used in this study.

Gene ID	Forward primer 5′-3′	Reverse primer 5′-3′	Purpose
**TDE0068**	CCGATGGAGACGGAGAATTA	CTCATCTGGGGCATCTAAGC	RT-PCR
**TDE0178**	TTGGCCGAGCAAGTTAAATC	CCGTTACCGTGTTGATACCC	RT-PCR
**TDE0345**	GTAGAAGTGCAAGCCGGTTC	TGCTGCCATCTCGTTCATAC	RT-PCR
**TDE0405**	TAGGCACGGATTCAAAGGTC	CGGCATAAGCAGACAAATCA	RT-PCR
**TDE0574**	CCCAGAGCCTTACCACGATA	GCAGCCCTTAAAAGCATCAG	RT-PCR
**TDE0606**	AGCCGAAAAACATGAGGACA	CTGCGTAGGATTACCGGAAC	RT-PCR
**TDE0670**	GCATTTTTACGCCGCTTTTA	AGTCCTTGGCTTGAGGGTTT	RT-PCR
**TDE0748**	ACAGCGTAAACGGAAGTGCT	GGCTTAACTCCCCAAACCAT	RT-PCR
**TDE0762**	TACTGGGTCCAATCCTCCAG	CGTCTTGTGCCGTAAAGGTT	RT-PCR
**TDE0937**	GGGAGATGAAGATGCCAAAA	CCATAAGGGGAAGACCTTGG	RT-PCR
**TDE1175**	GGGACAGGCAAAGAGCATAA	GGGCCTTGATCTGGGTAACT	RT-PCR
**TDE1382**	TAGTAAAAAGCCGCCGAAAC	TACCTGCCCTCCCTAATGTG	RT-PCR
**TDE1663**	TCGATCAGTTTACCGCACA	CTTCATCCTTTTGTGAATCCAG	RT-PCR
**TDE1795**	CATATTCAAGACCGCGTGAT	AGAAAAACATCCCGGTTTCC	RT-PCR
**TDE2123**	CAAGCCCAAAAGGGGACTAT	ATAAGGACGGCCACAACAAA	RT-PCR
**TDE2300**	ATACGGTTGGCTTGGTGTTC	TCCGCAGGAGAACCTAAAAA	RT-PCR
**TDE2327**	CCCGCAAATACAAGGAAGAA	CTTTTCGAGTTCGGGGATTT	RT-PCR
**TDE2480**	CCAGCTTTGCCGATTATGTT	ATGAGGAGATTGACGCAAGG	RT-PCR
**TDE2592**	AGGCGATCAAAACACAGGAA	CAACATAAGACCGCATCGTG	RT-PCR
**TDE2699**	GGAAGAAACCTGCACATCGT	GGGATTTTGCGTCGATAAGA	RT-PCR
**TDE0626**	AAAGACCGTAAAAGGCGAAGT		Operon analysis
**TDE0627**	TGAGTCTGCGGTGAAAGATG	AATCATTGAAACGGCTTCGT	Operon analysis
**TDE0628**	CATGTTTCGGCAAAAGACCT	TTATAACGGCCTCGGTTACG	Operon analysis
**TDE0629**	TAAGGGTACGGGACAAGTGC	CGCCGAAGATATCCTCAAAA	Operon analysis
**TDE0630**		AAAAATCTCGGGCTGACAAA	Operon analysis
**TDE0631**	TTGCTCATGAAATTGAAGATGAT	CGGCATTTAGCTGATCCAAT	Operon analysis
**TDE0934**	GTAAAACCGGATGCCGTAGA	CCGGCATATTTGTCGTGAAT	Operon analysis
**TDE0935**	AGTGCCAAAAAGGCACAAAT		Operon analysis
**TDE0936**	AACCATGCTAAAAGCCGAAT		Operon analysis
**TDE1173**		CTCCAACGTTTACCGCTGAT	Operon analysis
**TDE1174**	GGGATAAATGCATCAAGCAA	GATAAGTTCTCCGCCTGCTG	Operon analysis
**TDE1175**	GAAGATGCTCTTTCGGCAAC		Operon analysis
**TDE2123**	CAAGCCCAAAAGGGGACTAT		Operon analysis
**TDE2124**	CCCTTGAGCTTGAAGACGAC	GCAAGGCTGTTTCTTCAAGG	Operon analysis
**TDE2125**		AGCAAAGCCCAGCTTATGAA	Operon analysis
**TDE2479**		CAAGAAAGCCGTCAAGCAAT	Operon analysis
**TDE2480**	GATACGGCCTTCCCCATAAT	GATCGGTTTCGTCCACAACT	Operon analysis
**TDE2481**	TTCTCTCCCCTTGCCTTTTT		Operon analysis
**670 Flank 1**	CGGCAAAACCTTGTTGGATA	CGTTGCGGGCTAGCTAAAAGCGGCGTAAAAATGC	Cloning
**670 Flank 2**	CGCTTTTAGCTAGCCCGCAACGGTATAAAGGAAG	TATCATCAATTTCGCCATCG	Cloning
**Erm^R^**	GGATGATGGCTAGCCCGATAGCTTCCGCTATT	GGATGATGGCTAGCTTATTTCCTCCCGTTAAATAATA	Cloning

### Fluorescent cDNA preparation

For all microarray experiments, 5 µg of control or experimental RNA was combined with 10 µg of random hexamers and denatured at 70°C for 10 minutes prior to hybridization at 4°C for 10 minutes. The following was added at the final concentrations listed: 1× Superscipt buffer (Invitrogen), 100 µM DTT, 200 U of SuperScript® III Reverse Transcriptase (Invitrogen), 500 µM each of dATP, dCTP, and dGTP with 300 µM dTTP and 200 µM 5-(3-aminoallyl)-dUTP (Ambion). The reactions were then incubated in a thermocyler at 37°C for 10 minutes, 42°C for 1 hour 50 minutes, then 50°C for 10 minutes. RNA was then hydrolyzed in the presence of 0.1 M EDTA and 0.2 N NaOH at 65°C for 10 minutes. A final concentration of 0.3 M Hepes pH 7.5 was added to buffer the reactions. cDNA was further purified and concentrated using Microcon-30 filters (Millipore) and sodium bicarbonate (pH 9.0) was added to a final concentration of 0.1 M. Amersham mono-reactive Cy™3 and Cy™5 (GE Healthcare) dyes were diluted in DMSO and incubated with the corresponding cDNA samples in the dark for 1 hour at room temperature. Labeled cDNA was then purified with Wizard® SV Gel and PCR Clean-Up System (Promega) according to the manufacturer's protocol.

### Microarray hybridization and analysis

Microarrays were obtained through the NIAID's Pathogen Functional Genomics Resource Center, managed and funded by the Division of Microbiology and Infectious Diseases, NIAID, NIH, DHHS and operated by the J. Craig Venter Institute. Microarray experiments involving heat, oxygen and osmotic downshift were performed in five replicates, while the blood exposure experiment was performed in quadruplicate. Control cDNA was labeled with Cy3 and test cDNA was labeled with Cy5. Two arrays for each condition were used in dye-swapping experiments to address the possible effects of labeling bias. Freshly purified labeled test and control cDNA were combined prior to incubation with hybridization solution (Final concentrations: 3× SSC, 24 mM HEPES (pH 7.0), 0.225% SDS) at 95°C for 2 minutes. Samples were then evenly dispersed onto microarray slides with cover-slips by capillary action. Hybridization chambers were sealed and incubated at 48°C for 12 hours. Labeled arrays were washed twice with three sequential solutions for 10 minutes each. Solution 1 (low stringency) contained 2× SSC and 0.1% SDS and was heated to 55°C prior to washing the slides. Solution 2 (medium stringency) contained 0.1× SSC and 0.1% SDS. Solution 3 (high stringency) contained 0.1× SSC. Slides were briefly washed with water prior to drying and subsequent scanning with a Genepix 4000A scanner (MDS, Sunnyvale California).

Fluorescent intensities of each spot were calculated using Genepix Pro, version 6.0 (MDS). The program's morphological opening background subtraction was used to reduce noise and each array was normalized such that the average normalized ratio of medians was equal to one. The four in-slide replicates from each slide were combined and the resulting list containing a total of 20 (16 for blood exposure) replicates for each gene were normalized again such that the average normalized ratio of medians of each spot in the combined list was equal to one. The data sets were then subjected to statistical analysis using Significance Analysis of Microarray (SAM) software under an academic license from Stanford University. Delta values were chosen such that the false discovery rates were between 4–5% for each data set and were 0.727, 0.799, 0.748, and 0.58 for heat shock, oxygen shock, osmotic downshift and blood, respectively. Induced and repressed genes were extrapolated from significance lists generated by SAM by identifying the average ratio of median value of the replicates for each gene and selecting genes that had values either above 1.8 or below 0.55. Fold regulation shown in all tables is the average ratio of median value for each gene. Downregulated genes are presented as negative inverses of their respective decimal values. Data presented are in compliance with MIAME requirements. Microarray data were deposited on MIAMExpress (http://www.ebi.ac.uk/miamexpress/) with the accession number: E-MEXP-2656.

### Real-time quantitative PCR for microarray validation

**Figure 1:**
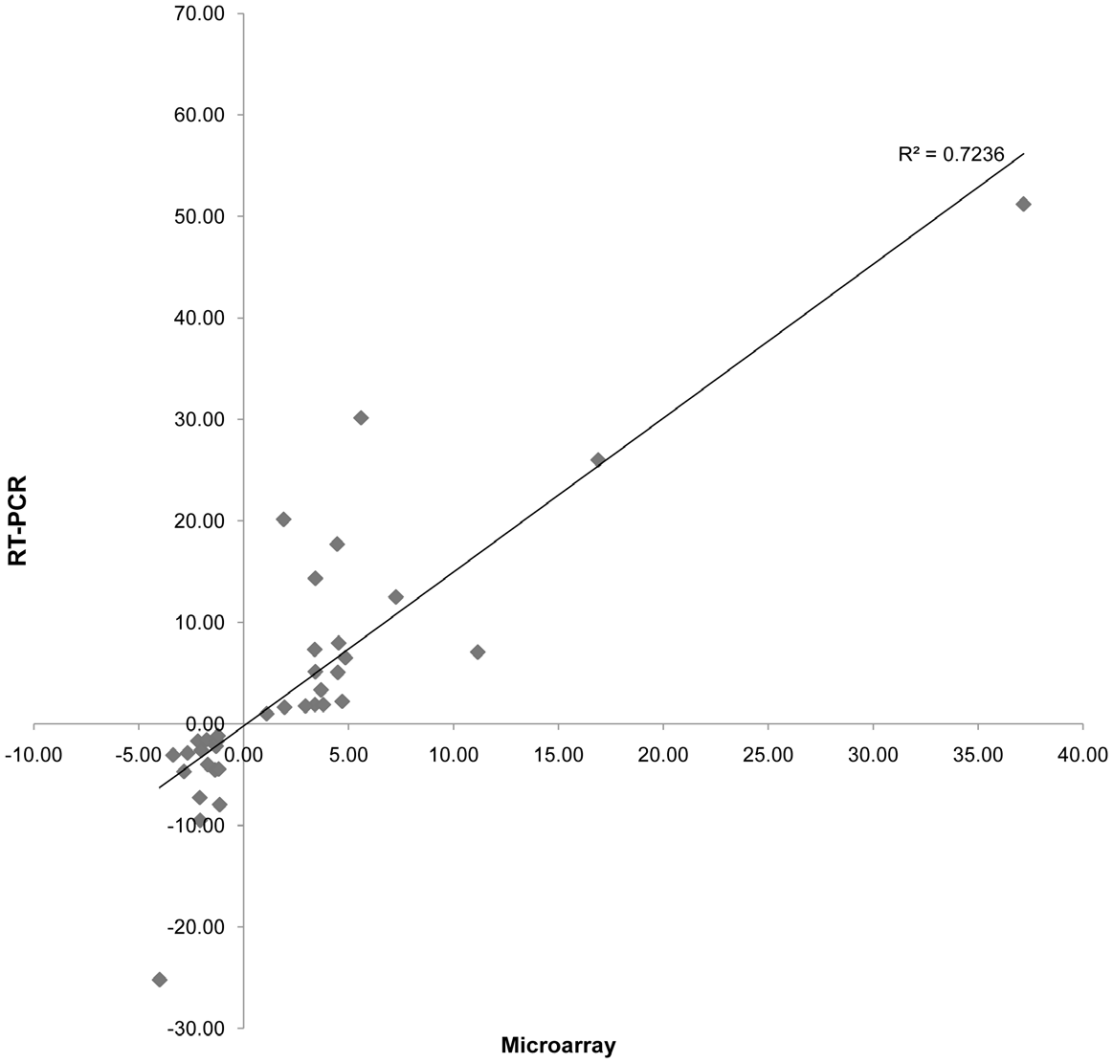
Correlation between microarray generated values and RT-PCR generated values. Expression values of 9 genes from each condition are compared. Trend line shown is the best-fit linear regression and the corresponding R^2^ value is indicated.

From a total list of 20 different genes, nine genes that represented various levels of microarray-predicted differential expression were selected from each environmental condition for real-time (RT) PCR analysis. PCR primers (Table 1) were designed using Primer3 [21] for each gene that specifically amplified between 90–120bp. RNA was reverse transcribed using the same protocol as described above, except 500 µM dTTP was used and no 5-(3-aminoallyl)-dUTP was added. cDNA was diluted 1∶50 in each PCR reaction. Quantitative PCR was performed in triplicates with a MyiQ Real-Time PCR Detection System (Biorad) and the accompanying program Biorad iQ5 using SYBR Green I (Invitrogen) fluorescence according to the manufacturer's protocol at a 1× concentration. Primers were used at a final concentration of 1 µM. Standard curves for each primer set were generated with iQ5 software using untreated control samples to allow quantification of transcript levels for each condition. The respective fold change was calculated by dividing the transcript abundance of each test sample by the transcript abundance of the corresponding control sample. Statistical significance of RT-PCR values was confirmed using the non-parametric Wilcoxon test. Intra-replicate comparison between microarray and RT-qPCR generated data for the same genes indicated a good correlation (Figure 1).

### Metabolic activity assay

Since *T. denticola* often exhibits a relatively poor and inconsistent colony forming efficiency, CellTiter-Blue™ Reagent (Promega) was employed as an alternative option to evaluate cell viability. This assay is based on the reduction of resazurin to resorufin as an indicator for metabolic activity which has been used previously to determine viability of *T. denticola* [22], [23]. Cultures of *T. denticola* were aliquoted into 15 ml tubes and exposed to heat, oxygen, a hypotonic solution, or blood in triplicate as described in the Strain and Growth Conditions section above. Over a 4 hour time period, 300 µl aliquots were taken every hour from each replicate, pelleted and resuspended in TYGVS with 20% CellTiter-Blue™ Reagent (Promega). Reactions were allowed to progress until the positive control appeared almost pink (usually about 2–5 minutes). Cells were again pelleted and 100 µl of the resulting supernatant was quickly removed from each tube, placed in a 96 well plate and absorbance readings were taken according to the manufacturer's protocol. Each experimental replicate is shown as a percentage of a comparable untreated control. To account for background optical density values of blood, the positive control for blood treatment was untreated cells that were immediately assayed for metabolic activity after addition of the same amount of blood used for treatment of the experimental sample. Statistical significance of data was confirmed using the Student's *t* test.

## Results

### Transcriptional profile in response to heat shock

**Figure 2:**
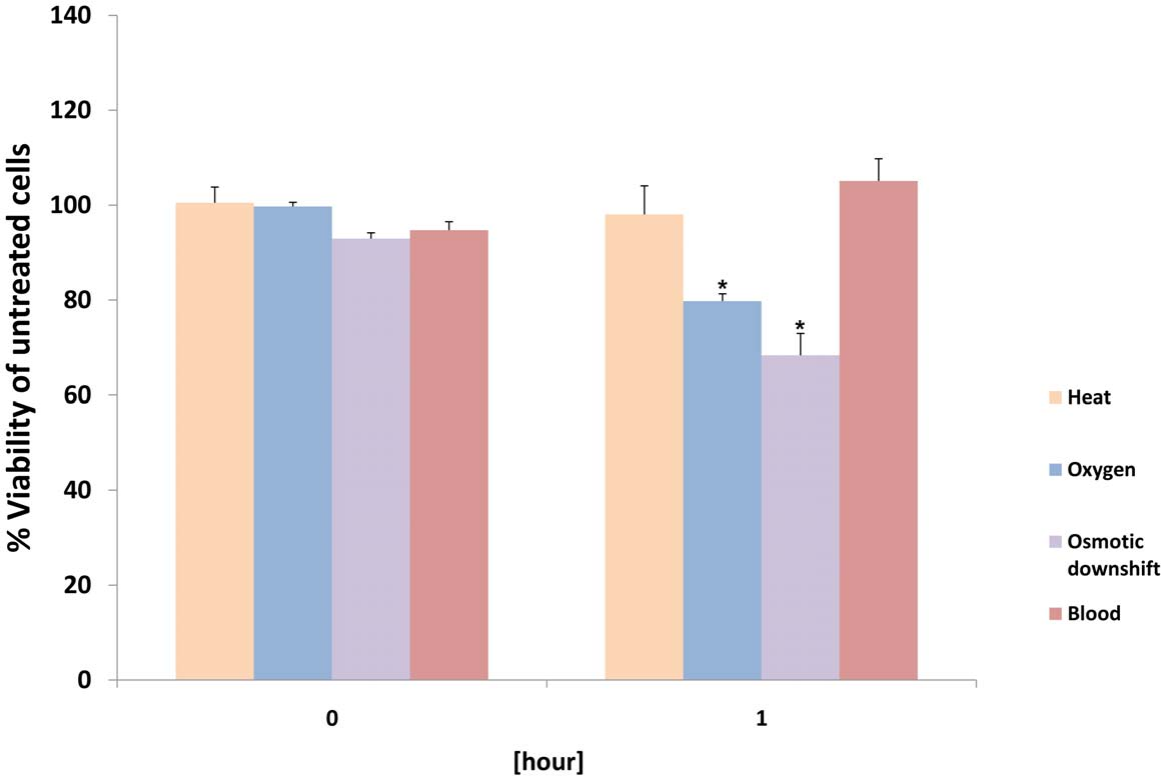
Time-course of viability for bacteria subjected to sudden changes in environmental conditions. Bacteria were exposed to each condition, as described in the materials and methods, for 1 hour. Samples were taken every hour and viability was analyzed using CellTiterBlue™ reagent. Data were plotted with untreated and heat killed cells serving as a reference points for 100% and 0%, respectively. *p<0.001.

Similar to previous studies of the *T. denticola* heat shock response [8], [9], heat stress was induced by shifting the incubation temperature from 37°C to 42°C for 1 hour. Given the poor colony forming efficiency of *T. denticola*, the impact of heat treatment on cell viability, was measured by determining metabolic activity as described in Material and Methods (Figure 2). Upon 1 hour heat shock, 112 of the 2,744 ORFs represented on the *T. denticola* microarray were found to be induced by at least 1.8 fold. By comparing the list of 112 induced genes with homologues of genes known to play a role during heat shock in other organisms [20], [21], we found that 14 of the 25 genes were upregulated upon heat stress (Table 2). Many of the ORFs annotated as chaperones exhibited an increase in expression under heat shock conditions, including *groES* [tde0934], *groEL* [tde1175], *grpE* [tde0627], *dnaK* [tde0628], *dnaJ* [tde0629] and *htpG* [tde2480]. ORFs predicted to encode heat shock-associated ATP-dependent proteases such as *lon* [tde0670], *clpA* [tde2124], *clpB* [tde2327], *clpS* [tde2123], *ftsH* [tde0470] and a possible *degP* orthologue [tde2300] were upregulated as well. A much smaller number of ORFs was repressed under heat shock conditions. The six downregulated genes encoded three hypothetical proteins, one protein with predicted enzymatic function, the cytoplasmic filament protein *cfpA* [tde0842] and *flgD* [tde2769], a predicted flagellar hook assembly scaffolding protein. The complete list of up- and downregulated genes is available in Table S1.

**Table 2:** Upregulation of predicted heat shock genes.

		Ortholog			Fold change		
Locus	*T. denticola*	*B. subtilis*	*E. Coli*	Protein description	Heat	Oxygen	Osmotic downshift
TDE0470	*ftsH*	*ftsH*	*ftsH*	cell division protein FtsH	2.8	11.5	5.3
TDE0627	*grpE*	*grpE*	*grpE*	co-chaperone protein GrpE	3.4	8.4	4.3
TDE0628	*dnaK*	*dnaK*	*dnaK*	chaperone protein DnaK	5.6	9.7	3.7
TDE0629	*dnaJ*	*dnaJ*	*dnaJ*	chaperone protein DnaJ	3.8	2.0	
TDE0670	*lon*	*lonA*	*lon*	ATP-dependent protease La	4.1	8.0	
TDE0934	*groES*	*groES*	*groES*	chaperonin, 10 kDa	2.2	2.2	
TDE0937		*∼sigA*	*∼rpoD*	RNA polymerase sigma-70 factor family protein	2.8	21.8	14.0
TDE1029			*ibpB*	HSP20/alpha crystallin family protein	9.5	8.5	6.7
TDE1175	*groEL*	*groEL*	*groEL*	chaperonin, 60 kDa	4.3	10.2	4.7
TDE1210	*HslV*	*clpQ*	*clpQ*	heat shock protein HslVU, ATP-dependent protease			
TDE1211	*hslU*	*clpY*	*clpY*	heat shock protein HslVU, ATPase subunit HslU			
TDE1332	*hflK*		*hslY*	hflK protein, putative			
TDE1488	*gap*	*gapA*	*gapA*	glutamate racemase, putative			
TDE1672	*clpP-1*	*clp-P*	*clp-P*	ATP-dependent Clp protease, proteolytic subunit ClpP			
TDE1673	*clpX*	*clp-X*	*clp-X*	ATP-dependent Clp protease, ATP-binding subunit ClpX			
TDE1966	*htrA-1*	*htrA/htrB*		trypsin domain/PDZ domain protein		3.7	
TDE1968	*ftsJ*		*ftsJ*	cell division protein FtsJ			
TDE2123	*clpS*	*clpS*	*clpS*	ATP-dependent Clp protease adaptor protein ClpS	4.0	24.0	4.2
TDE2124	*clpA*	*clpC*	*clpA*	ATP-dependent Clp protease, ATP-binding subunit ClpA	4.3	9.4	2.1
TDE2300		*htrA/htrB*	*degP*	trypsin domain/PDZ domain protein	2.2	6.3	
TDE2327	*clpB*	*clpB*	*clpB*	ATP-dependent Clp protease, ATP-binding subunit ClpB	3.7	10.0	
TDE2388	*clpP-2*	*clpP*	*clpP*	ATP-dependent Clp protease, proteolytic subunit ClpP		3.0	
TDE2480	*htpG*	*htpG*	*htpG*	chaperone protein HtpG	4.7	3.3	
TDE2554		*(yacC)*	*hsp33*	chaperonin, 33 kDa family			
TDE2750		*(ynbA)*	*hslX*	GTP-binding protein HflX, truncation			

### Transcriptional profile in response to oxygen shock

In order to investigate the transcriptional response of to oxygen, another important environmental stress for this anaerobic oral species, *T. denticola* cells were exposed to oxygen for 1 hour as described in Material and Methods. Under these conditions metabolic activity was decreased to about 80% of the untreated control sample (Figure 2). Applying this condition to microarray analysis resulted in the induction of 211 and repression of 15 genes. Seven of the upregulated ORFs encode homologues of proteins for detoxification of reactive oxygen species including a peroxiredoxin [tde0011], several thioredoxins [tde0238, tde0743, and tde0744], a ferritin [tde0449], a rubredoxin [tde1052], and a desulforedoxin [tde1754]. The 1 hour oxygen exposure also induced the same set of chaperones that was upregulated during heat stress. Furthermore, transcript levels for two additional predicted proteases, *htrA-1* [tde1966] and *clpP* [tde2388], were elevated under oxygen stress, while no difference was observed for the ORF encoding the predicted redox-regulated chaperone *hsp33* [tde2554]. The complete list of affected genes is available in Table S1. Other upregulated genes coded for 11 transcriptional regulators including seven that appear to be unique to oxygen stress (Table 3), 16 predicted transport proteins (Table S2), eight proteins involved in motility or chemotaxis, 75 proteins for other cellular processes and 85 proteins of unknown function. The 15 downregulated transcripts are annotated as encoding eight proteins with enzymatic or homeostatic functions, four transporter proteins, and three hypothetical proteins.

**Table 3:** Changes in expression profiles of putative transcriptional regulators.

			Fold change			
Locus	Gene	Protein Description	Heat	Oxygen	Osmotic downshift	Blood
TDE0070		RNA polymerase sigma-70 factor, region 2 family			−1.8	
TDE0082		transcriptional regulator, MerR family				1.8
TDE0127		DNA-binding protein		1.9		
TDE0264		ribbon-helix-helix protein, CopG family		2.9		
TDE0332		transcriptional regulator, TetR family		3.2		
TDE0630		sigma factor regulatory protein, putative	2.1			
TDE0660		transcriptional regulator, putative				2.6
TDE0791	*rpoA*	DNA-directed RNA polymerase, alpha subunit				−2.1
TDE0937		RNA polymerase sigma-70 factor family protein	2.8	21.8	14.0	
TDE1222		iron-dependent transcriptional regulator		2.7	2.2	
TDE1346		RNA polymerase sigma-70 factor family protein			−1.8	
TDE1382		transcriptional regulator, ArsR family		8.3	9.3	−3.1
TDE1601		transcriptional regulator, TetR family				2.1
TDE1647		DNA-binding protein		2.0	1.9	
TDE1670		transcriptional regulator, MarR family		2.3		
TDE1953		transcriptional regulator, TetR family				−2.1
TDE2083		anti-anti-sigma factor		2.1		
TDE2324		DNA-binding response regulator		1.8		
TDE2420		DNA-directed RNA polymerase, beta′ subunit, putative				−2.0
TDE2650		transcriptional regulator, putative		2.5		

### Transcriptional profile in response to osmotic downshift

Saliva, crevicular fluid, and tissue, represent a range of osmotic conditions experienced by oral bacteria. Since oral spirochetes predominantly reside in the gingival pocket which is comprised of crevicular fluid and gingival tissue, transition into hypotonic fluids such as saliva [13] would require adaptation to this downshift in environmental osmolarity. Similar to the analysis of the heat shock and oxygen stress responses, *T. denticola* cells were subjected to osmotic downshift conditions and metabolic activity was measured to evaluate cell viability (Figure 2). The experimental conditions chosen for this study yielded induction of 125 and repression of 98 genes, respectively. The 125 upregulated genes encoded 11 putative transport proteins, seven chaperones and proteases, four potential transcription regulators, 54 proteins involved in other cellular processes, two chemotaxis proteins and 47 proteins of unknown function. The downregulated genes coded for two transcriptional regulators, 13 proteins involved in transport, two chemotaxis proteins, 54 proteins involved in other cellular processes and 27 proteins of unknown function (Table S1).

### The core stress response genes of *Treponema denticola*

**Figure 3:**
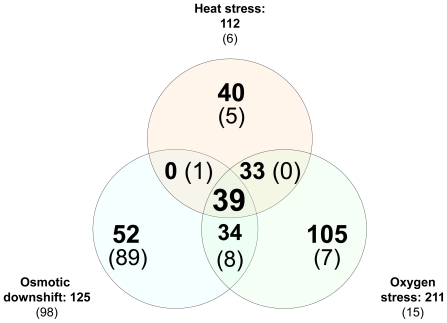
Overlapping stress response genes. Genes induced (in bold) and repressed (in parenthesis) from each stress were overlaid to identify the number of overlapping genes.

The different transcriptional responses of *T. denticola* to the stress conditions tested in this study (heat, oxygen exposure and osmotic downshift) were compared to identify a core set of genes regulated upon these environmental challenges (Figure 3). A total of 39 genes were found to be induced under all of the three different stresses tested (Table 4), while no such overlap was observed among the repressed genes. Not surprisingly, included among the 39 upregulated genes are several of the ubiquitous chaperones and proteases that are conserved throughout the tree of life [24]. This finding underscores the roles of these chaperones and proteases in general stress response mechanisms, as opposed to being specific to an individual stress. Additionally, the σ-70 homologue encoded by tde0937 emerged as a potential global regulator of the core stress response since it was induced under all stress conditions tested. The gene encoding the methyl-accepting chemotaxis protein *dmcB* is significantly induced under all three stress conditions tested, indicating a potential role of chemotaxis during the stress response of *T. denticola*. We also found that the response to heat shock and oxygen exposure shared 33 differentially regulated genes. A set of 34 induced and eight repressed ORFs overlapped between osmotic downshift and oxygen stress, while only one repressed gene was shared between osmotic downshift and heat shock, likely reflecting the disparate stimuli (Figure 3).

**Table 4:** Upregulation of shared stress response genes.

			Fold change			
Locus	Gene	Protein description	Heat	Oxygen	Osmotic downshift	Blood
TDE0160		hypothetical protein	1.8	6.2	3.2	
TDE0345	*dmcB*	methyl-accepting chemotaxis protein DmcB	2.9	5.1	5.7	
TDE0351	*ldh*	L-lactate dehydrogenase	1.9	2.0	1.9	1.8
TDE0386		ABC transporter, periplasmic substrate-binding protein	2.1	9.0	2.9	2.6
TDE0449		ferritin, putative	3.0	8.3	4.0	3.4
TDE0468		conserved hypothetical protein	1.8	2.8	2.5	
TDE0470	*ftsH*	cell division protein FtsH	2.8	11.5	5.3	
TDE0627	*grpE*	co-chaperone protein GrpE	3.4	8.4	4.3	2.1
TDE0628	*dnaK*	chaperone protein DnaK	5.6	9.7	3.7	1.8
TDE0667	*sufB*	FeS assembly protein SufB	4.5	10.6	4.2	
TDE0709	*msrA*	methionine-R-sulfoxide reductase	3.9	6.8	4.4	
TDE0738		conserved hypothetical protein	2.7	7.1	5.6	
TDE0832		hypothetical protein	2.7	10.4	2.0	
TDE0937		RNA polymerase sigma-70 factor family protein	2.8	21.8	14.0	
TDE1028		hypothetical protein	2.9	16.6	8.7	
TDE1029		Hsp20/alpha crystallin family protein	9.8	8.5	6.7	
TDE1051		conserved hypothetical protein	3.6	8.4	4.8	
TDE1052		rubredoxin	2.0	3.8	1.9	
TDE1103		hypothetical protein	3.2	4.8	3.2	
TDE1175	*groEL*	chaperonin, 60 kDa	4.3	10.2	4.7	
TDE1296		ribosomal subunit interface protein, putative	2.5	5.6	2.0	
TDE1384	*cadA*	cadmium-translocating P-type ATPase	2.0	2.3	1.8	
TDE1618		conserved hypothetical protein	1.8	11.3	4.0	4.3
TDE1663		OmpA family protein	2.0	5.6	5.2	2.5
TDE1664		conserved domain protein	2.0	4.6	2.9	3.1
TDE1693		hypothetical protein	1.9	4.5	3.3	
TDE1694		hypothetical protein	2.5	5.0	2.5	
TDE1737		hypothetical protein	2.7	2.3	2.1	
TDE1754		desulfoferrodoxin/neelaredoxin	3.6	6.3	3.2	2.0
TDE1926		membrane protein, putative	3.9	6.5	2.1	
TDE2123		ATP-dependent Clp protease adaptor protein ClpS	4.0	24.0	4.2	1.8
TDE2124	*clpA*	ATP-dependent Clp protease, ATP-binding subunit ClpA	4.3	9.4	2.1	
TDE2223		hypothetical protein	1.8	3.4	2.6	
TDE2226		ABC transporter, substrate-binding protein, putative	2.7	2.6	4.6	
TDE2350		lipoprotein, putative	1.8	5.2	2.1	
TDE2372		conserved hypothetical protein	3.0	3.7	4.6	2.6
TDE2373		precorrin-6Y C5,15-methyltransferase, putative	4.6	6.0	3.2	
TDE2590		hypothetical protein	4.0	9.0	4.2	3.7
TDE2591		rhodanese-like domain protein	3.8	6.5	3.6	2.5

To investigate co-transcription of stress response genes and neighboring ORFs that are organized in the same direction, we performed a limited operon analysis of several genes using reverse transcription-PCR. We examined cDNA from oxygen shock, since this condition exhibited the highest level of induction for most of the genes of interest among the stresses tested. PCR was performed with various primer combinations homologous to the respective neighboring genes (Table 1 – operon analysis). Co-expression was assumed if bands were detectable upon agarose gel electrophoresis and the corresponding results are summarized in Table 5. The chaperones *grpE* [tde0627], *dnaK* [tde0628] and *dnaJ* [tde0629] are co-expressed. Consistent with our microarray results, additional downstream ORFs, beginning with tde0630, that are organized in the same direction do not appear to be part of the same transcriptional unit. The gene for *groES* [tde0934] and the downstream ORF [tde0935], encoding a small hypothetical protein, are co-transcribed. Similarly, *clpS* [tde2123] was confirmed to be organized in an operon with *clpA* [tde2124], separate from additional downstream ORFs. Finally, *groEL* [tde1175] or *htpG* [tde2480] were not co-expressed with their respective up- or downstream genes. Since we only examined cDNA derived from cells exposed to oxygen stress, we cannot rule out the possibility that co-expression with adjacent genes occurs under different experimental conditions.

**Table 5:** Operon analysis.

Co-expression tested	Co-expression detected	Gene names
TDE0626 - TDE0633	TDE0627 - TDE0629	*grpE, dnaK, dnaJ*
TDE0934 - TDE0936	TDE0934 - TDE0935	*groES*, hypothetical protein
TDE2123 - TDE2125	TDE2123 - TDE2124	*clpS*, *clpA*
TDE1173 - TDE1175	TDE1175	*groEL*
TDE2479 - TDE2481	TDE2480	*htpG*

### Transcriptional profile in response to blood exposure

**Figure 4:**
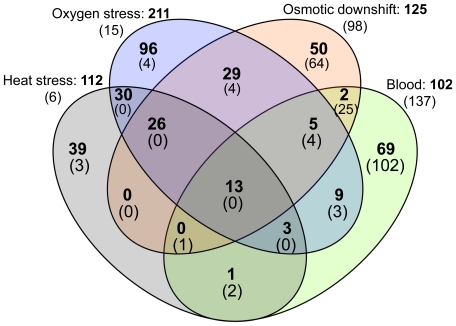
Genes overlapping between stresses and blood. Genes induced (in bold) and repressed (in parenthesis) from each condition were overlaid to identify the number of overlapping genes.

*T. denticola* was incubated in the presence of 20% sheep's blood to determine the transcriptional response to this important environmental factor. Metabolic activity had slightly increased at the 1 hour time point (Figure 2) and microscopic examination showed that *T. denticola* cells partially aggregate upon blood exposure. Microarray analysis revealed the induction of 102 genes and repression of 137 genes. Comparison of these “blood”-regulated genes with the ones regulated by the different stresses tested in this study (Figure 4), indicated association of about 32% (33 out of the 102) of the upregulated ORFs with at least one of the stress conditions tested; 13 of which were upregulated in all the conditions tested (Table 4 - Blood). The 69 uniquely blood-stimulated genes represent the following classes of genes: 41 of the genes identified were hypothetical or conserved hypothetical genes, three genes encode predicted transporter proteins, three genes are annotated as transcriptional regulators and 22 genes are predicted to be involved in other cellular processes (Table S1). This list of genes likely contains genes relevant for survival in blood, pathogenesis or aggregation. Genes that were only found to be repressed in the presence of blood are predicted to encode four transcriptional regulators, 13 proteins involved in transport, five motility-related proteins, 61 proteins for other cellular processes, 16 proteins of unknown function and three genes previously identified as potential virulence factors: *prtP*, *prcA*, and *msp* [17], [25], [26], [27].

## Discussion

### Core stress response

Oral bacteria are frequently exposed to a variety of diverse environmental stresses and respond by producing remedial proteins such as heat shock proteins. Recent findings indicate that some of these stress proteins may play roles in oral microbial pathogenesis [28], [29]. Despite the obvious relevance of stress-induced proteins for survival and virulence during pathogenic processes, very few studies have investigated the cellular responses of periodontal pathogens to adverse conditions. Comparison of the transcriptional response to the stresses tested in this study (heat shock, oxygen exposure and osmotic downshift) allowed identification of a set of core stress response genes that is not specific to any single stimulus (Table 4). This core stress response included induction of the chaperones *grpE* [tde0627], *dnaK* [tde0628], *groEL* [tde1175], *ibpB* (an HSP20 homolog) [tde1029], and the proteases *ftsH* [tde0470], *clpS* [tde2123], *clpA* [tde2124] and *degP* [tde 2300]. Reduction of the analysis criteria to heat shock and oxygen exposure, which are more likely to cause extensive protein damage, expands the list of genes to the chaperones *dnaJ* [tde0629], *groES* [tde0934], *htpG* [tde2480] as well as the proteases *lon* [tde0670] and *clpB* [tde2327] all of which have been found in other organisms to be responsive to the cellular accumulation misfolded proteins [30].

In addition to above ubiquitous components of cellular stress responses, a predicted σ^70^-factor [tde0937] was induced under all stress conditions tested (Table 3). Since homologs of RpoS and σ^B^, the global regulators of some stress responses in *E. coli* and *Bacillus subtilis* [31], [32], respectively, are missing in *T. denticola*, this σ-factor could represent a regulator of stress responses. Recently, the same σ^70^-factor [tde0937] was reported to be downregulated in *T. denticola* biofilms [33]. The ArsR family transcriptional regulator, [tde1382], was upregulated upon osmotic downshift and oxygen stress, but downregulated during blood exposure (Table 3). ArsR family transcriptional regulators were found to act as transcriptional repressors for operons that regulate metal concentrations in the cell [34]. Given that this gene induced under high iron concentration (blood) and repressed under conditions of lower iron concentration (osmotic downshift), this ArsR could be involved in regulating intracellular metal concentrations. When comparing *arsR* expression profiles with predicted transporters, we found that homologues of *troB* [tde1225] and *troA* [tde1226] followed the same expression pattern, suggesting a possible connection. A notable detail of the core stress response was the upregulation of the chemosensor, *dmcB* [tde0345], among all stresses tested (Table 4). DmcB has previously been implicated in motility/chemotaxis behavior and tissue invasion of *T. denticola* [35], [36]. The induction of *dmcB* expression upon every condition tested suggests a role of chemotaxis in evading adverse conditions.

### Heat shock response

While previous studies of the heat shock response in *T. denticola* only demonstrated the presence of DnaK and GroEL, the microarray-based expression profiling approach in this study revealed a more comprehensive response profile. In addition to the apparent core stress response genes, multiple ATPases, transporters and hypothetical proteins were induced during heat shock in addition to the core stress response genes, while very few genes were repressed (Tables S1 and S2). The putative transcriptional regulator [tde0630] that was only induced during heat stress (Table 3) could control expression of some of the 42 genes that were specifically altered under this condition. Additional transcriptional regulators might be relevant at earlier time points which were not tested in this study.

### Oxygen stress response

With 226 differentially expressed genes, oxygen stress resulted in the most complex cellular response among the environmentala changes examined in this study. A recent study showed that *T. denticola* removes oxygen from aerobic environments to generate anaerobic microenvironments [37] and possesses oxidoreductase activity [38]. In response to oxygen stress, other anaerobic oral bacteria, including many oral *Streptococcus* species, make use of various NADH-oxidases, NADH-peroxidases, superoxide dismutases, glutathione reductases and pyruvate oxidases to metabolize oxygen into less harmful derivatives [39]. Based on our microarray analysis *T. denticola* appears to employ different mechanisms for detoxification upon extended oxygen exposure since its NADH-oxidase [tde0096] and glutathione peroxidase homologs [tde1729] were not induced. Instead, a number of redoxins and proteins involved in iron homeostasis were differentially regulated (Table S1). Several of the ORFs suggested to be involved in the oxidative stress response [12], were significantly induced under our experimental conditions [tde0011, tde1754, tde0238, tde2119], while others could not be confirmed. Most strikingly, transcript levels for the peroxiredoxin [tde0011] were 38.6-fold higher than untreated controls (Table S1).

In the periodontal pathogen *P. gingivalis*, a peroxiredoxin (homologous to tde0011), a thioredoxin (homologous to tde0743 and tde0744), and a ferritin (homologous to tde0449) are under the control of OxyR [40], [41]. Since the *T. denticola* genome does not contain an OxyR homologue, differential regulation of these genes could be controlled by one of the several transcriptional regulators that are specifically induced under oxygen stress conditions (Table 3) or by a yet unidentified transcriptional regulator. Surprisingly, the Hsp33 homologue [tde2554], a chaperone whose activity in other anaerobic species is regulated by a redox-switch involving a zinc-center [42] was not induced.

### Response to osmotic downshift

The transition from the isotonic gingival crevice to hypotonic saliva represents an osmotic downshift that has not been extensively studied in oral bacteria. In other organisms mechano-sensitive channels and aquaporins have been implicated in osmoregulation of cell turgor upon osmotic downshift [14], [15]. Consistent with the observation that mechano-sensitive channels are generally constitutive and primarily respond via conformational changes, the two mechano-sensitive transporter homologs of *T. denticola*, TDE2323 and TDE2295, were not induced at the 1 hour time point tested in this study. Homologues to osmo-regulated periplasmic glucans that certain gram-negative species employ as an additional layer of protection [16], are absent from the *T. denticola* genome [12].

While a number of ORFs are exclusively induced upon osmotic downshift, a considerable overlap with genes that are differentially regulated upon oxygen exposure, including chaperones, proteases, and proteins involved in protection from oxygen damage, was apparent (Tables S1 and S2). Certain small hypothetical proteins also appear to be specifically induced under these stress conditions but not upon exposure to heat.

### Response to blood exposure

Considering that *T. denticola* resides in infected gingival tissue prone to bleeding and has been implicated in atherosclerotic plaque buildup, adaptation to blood-rich environments is likely. Even though many pathogens are disseminated in the host via the blood stream, few studies have examined bacterial responses to the presence of blood. Among the 171 specifically altered genes in the blood response, few are part of the core stress response identified for *T. denticola* (Table 4). Consistent with the notion that blood may not comprise a severe stress for *T. denticola*, cell viability was not affected upon extended (up to 4 hours) of blood exposure, while all other conditions lead to a significant reduction during the same time period (data not shown). Three genes encoding putative transcriptional regulators (Table 3) and three genes associated with transport (Table S2) were upregulated, while transcripts for the major surface protein [tde0405] and numerous subunits of the outer membrane-associated protease, Dentilisin [tde0761 and tde0762], were downregulated. This is consistent with the previously observed downregulation of surface antigens as a possible immune-evasion strategy [43] since a recent finding that identified both Dentilisin and the major surface protein as key targets for human antibodies [44].

### Concluding remarks

In this report, we analyzed expression profiles of *T. denticola* in response to several changes in environmental conditions that are relevant for the survival of this suspected periodontal pathogen in the oral cavity. In addition to analyzing responses to individual stresses, we compiled a possible core stress response. These data could prove valuable for future studies investigating potential pathogenic and immune evasion mechanisms of *T. denticola*.

## Supporting Information

Table S1 Combined microarray data set. Average ratio of median for each data point is shown for genes altered by more than 1.8 fold in all conditions. Numbers highlighted in grey indicate downregulation. The overlapping conditions column indicates the number of conditions tested that each gene is altered in.
(0.11 MB XLS)

Table S2 Altered expression of putative transporter genes. Expression of 50 transporter genes was altered. Numbers highlighted in grey indicate downregulation.
(0.08 MB DOC)
