# Supplementary material for: Transcriptional Profiles of Treponema denticola in Response to Environmental Conditions
Source: PLoS One. 2010 Oct 27;5(10):e13655. doi: 10.1371/journal.pone.0013655 (PMC2965109; doi:10.1371/journal.pone.0013655)
Supplement: Table S2 — Altered expression of putative transporter genes. Expression of 50 transporter genes was altered. Numbers highlighted in grey indicate downregulation. (0.08 MB DOC) [file pone.0013655.s002.doc]

**Supplemental Table 2.** Changes in expression profiles of putative transport genes.

|  |  |  | **Fold change** | | | |
| --- | --- | --- | --- | --- | --- | --- |
| **Locus** | **Gene** | **Protein Description** | **Heat** | **Oxygen** | **Osmotic**  **downshift** | **Blood** |
| TDE0045 |  | ABC transporter, ATP-binding protein |  |  | 1.8 |  |
| TDE0144 |  | thiamine ABC transporter, permease protein |  | 2.2 |  |  |
| TDE0243 |  | ABC transporter, ATP-binding protein |  |  | 2.1 |  |
| TDE0245 |  | ABC transporter, ATP-binding/permease protein |  |  | -1.9 |  |
| TDE0273 |  | ABC transporter, ATP-binding/permease protein |  |  | -1.9 |  |
| TDE0274 |  | ABC transporter, ATP-binding/permease protein |  |  | -1.9 |  |
| TDE0359 |  | ABC transporter, ATP-binding/permease protein |  | 2.5 |  |  |
| TDE0373 |  | ABC transporter, ATP-binding/permease protein |  |  |  | 2.1 |
| TDE0386 |  | ABC transporter, periplasmic substrate-binding protein | 2.1 | 9.0 | 2.9 | 2.6 |
| TDE0394 |  | oligopeptide/dipeptide ABC transporter, permease protein |  |  |  | 1.8 |
| TDE0475 |  | ABC transporter, ATP-binding protein | 2.0 | 2.7 |  |  |
| TDE0535 |  | ABC transporter, ATP-binding protein |  |  |  | -2.4 |
| TDE0637 |  | oligopeptide/dipeptide ABC transporter, ATP-binding protein |  |  |  | -1.8 |
| TDE0680 | *secF* | protein-export membrane protein SecF |  |  | -1.9 | -1.9 |
| TDE0681 | *secD* | protein-export membrane protein SecD |  | -1.8 | -2.1 | -2.6 |
| TDE0682 | *yajC* | preprotein translocase, YajC subunit |  |  |  | -1.8 |
| TDE0758 |  | iron compound ABC transporter, periplasmic iron compound-binding protein, putative |  |  |  | -2.0 |
| TDE0787 | *secY* | preprotein translocase, SecY subunit |  |  |  | -4.5 |
| TDE0922 |  | ABC transporter, ATP-binding/permease protein |  |  | 2.3 |  |
| TDE0923 |  | ABC transporter, ATP-binding/permease protein |  | 1.9 |  |  |
| TDE0924 |  | ABC transporter, ATP-binding/permease protein |  | 2.3 |  |  |
| TDE0954 |  | branched-chain amino acid ABC transporter, permease protein |  |  | -1.8 |  |
| TDE0984 |  | oligopeptide/dipeptide ABC transporter, permease protein, putative |  | -2.1 | -1.8 |  |
| TDE0985 |  | oligopeptide/dipeptide ABC transporter, periplasmic peptide-binding protein, putative |  |  |  | -3.0 |
| TDE0986 |  | oligopeptide/dipeptide ABC transporter, ATP-binding protein |  |  |  | -2.6 |
| TDE1068 |  | oligopeptide/dipeptide ABC transporter, ATP-binding protein |  |  |  | -2.1 |
| TDE1069 |  | oligopeptide/dipeptide ABC transporter, permease protein |  |  |  | -1.8 |
| TDE1070 |  | oligopeptide/dipeptide ABC transporter, permease protein |  |  | -2.0 | -2.3 |
| TDE1073 |  | oligopeptide/dipeptide ABC transporter, permease protein |  | -1.9 |  |  |
| TDE1180 |  | iron compound ABC transporter, periplasmic iron compound-binding protein |  |  | 1.8 |  |
| TDE1183 |  | ABC transporter, ATP-binding protein | 1.9 |  |  |  |
| TDE1223 | *troD* | zinc ABC transporter, permease protein |  | 2.4 | 2.6 |  |
| TDE1224 | *troC* | zinc ABC transporter, permease protein |  | 2.3 |  |  |
| TDE1225 | *troB* | zinc ABC transporter, ATP-binding protein |  | 3.6 | 3.9 | -2.0 |
| TDE1226 | *troA* | zinc ABC transporter, periplasmic zinc-binding protein |  | 3.6 | 2.3 | -1.9 |
| TDE1274 |  | oligopeptide/dipeptide ABC transporter, permease protein |  | -1.9 | -2.0 |  |
| TDE1516 |  | ABC transporter, ATP-binding protein, putative |  |  | -1.8 |  |
| TDE1653 |  | ABC transporter, ATP-binding protein, authentic frameshift |  |  | -2.0 |  |
| TDE1879 |  | ABC transporter, ATP-binding protein, authentic frameshift |  |  | -1.8 |  |
| TDE1898 | *secA* | preprotein translocase, SecA subunit | 2.0 | 2.3 |  |  |
| TDE2063 | *feoB* | ferrous iron transport protein B |  |  |  | 2.0 |
| TDE2226 |  | ABC transporter, substrate-binding protein, putative | 2.7 | 2.6 | 4.6 |  |
| TDE2233 |  | iron compound ABC transporter, permease protein, putative |  |  |  | -2.4 |
| TDE2234 |  | iron compound ABC transporter, periplasmic iron compound-binding protein, putative |  |  |  | -2.4 |
| TDE2284 |  | efflux transporter, RND family, MFP subunit | 1.8 | 2.0 |  |  |
| TDE2365 |  | high-affinity branched-chain amino acid ABC transporter, permease protein | 1.8 |  |  |  |
| TDE2511 |  | ABC transporter, ATP-binding/permease protein |  | 1.9 |  |  |
| TDE2625 |  | ABC transporter, ATP-binding/permease protein | 2.1 |  |  |  |
| TDE2626 |  | ABC transporter, ATP-binding/permease protein |  | 2.1 | 2.9 |  |
| TDE2649 |  | ABC transporter, ATP-binding protein |  |  | -1.8 |  |
